# Supplementary material for: Profiling serum antibodies with a pan allergen phage library identifies key wheat allergy epitopes
Source: Nat Commun. 2021 Jan 22;12:379. doi: 10.1038/s41467-020-20622-1 (PMC7822912; doi:10.1038/s41467-020-20622-1)
Supplement: Supplementary file 1 — Supplementary Information [file 41467_2020_20622_MOESM1_ESM.pdf]

Supplemental Information:

Profiling Serum Antibodies with a Pan Allergen Phage Library Identifies Key Wheat Allergy Epitopes

Daniel R. Monaco[1], Brandon M. Sie[2], Thomas R. Nirschl[1], Audrey C. Knight[1], Hugh A. Sampson[3], Anna Nowak-Wegrzyn[3], Robert A. Wood[4], Robert G. Hamilton[5], Pamela A. Frischmeyer-Guerrerio[6*] & H. Benjamin Larman[1*]

[1]Institute for Cell Engineering, Division of Immunology, Department of Pathology, Johns Hopkins School of Medicine, Baltimore, MD, USA.

[2]Bioinformatics and Integrative Genomics PhD Program, Harvard Medical School, Boston, MA, USA.

[3]Icahn School of Medicine at Mount Sinai, New York, NY, USA.

[4]Johns Hopkins University School of Medicine, Johns Hopkins Hospital, Baltimore, MD, USA.

[5]Division of Allergy and Clinical Immunology, Department of Medicine, and Department of Pathology, Johns Hopkins University School of Medicine, Baltimore, MD 21205, USA.

[6]The Laboratory of Allergic Diseases, National Institutes of Allergy and Infectious Diseases, Bethesda, MD, USA.

[*] address correspondence to hlarman1@jhmi.edu or pamela.guerrerio@nih.gov

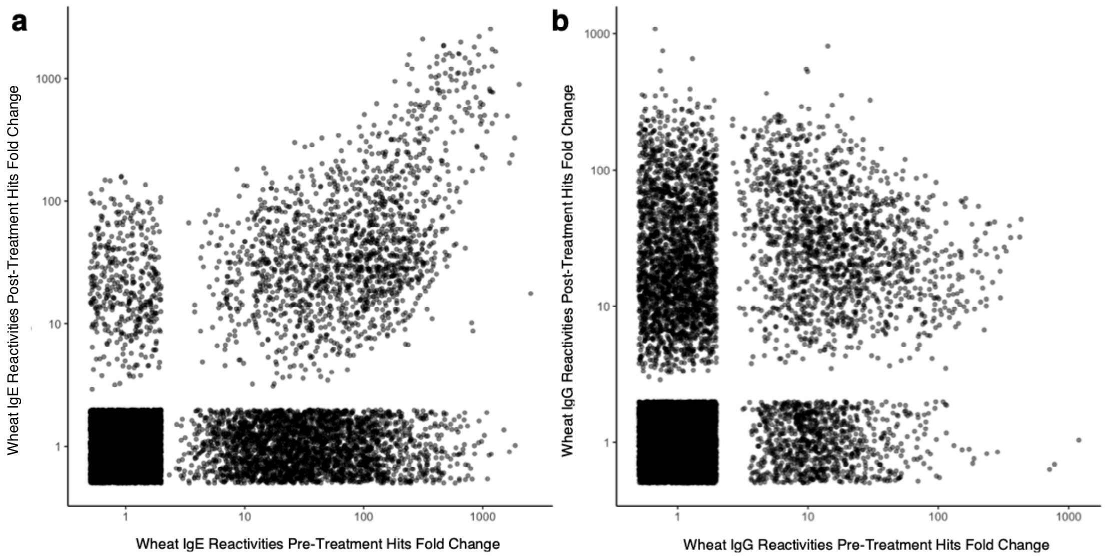

Supplemental Figure 1. Wheat Oral Immunotherapy Trial Isotype Specific Results. **a**. Scatterplot of IgE reactivities to wheat peptides identified in Fig 4, comparing data from before and after active WOIT treatment. **b**. Scatterplot of IgG reactivities to wheat peptides identified in Fig 4, comparing data from before and after active WOIT treatment.

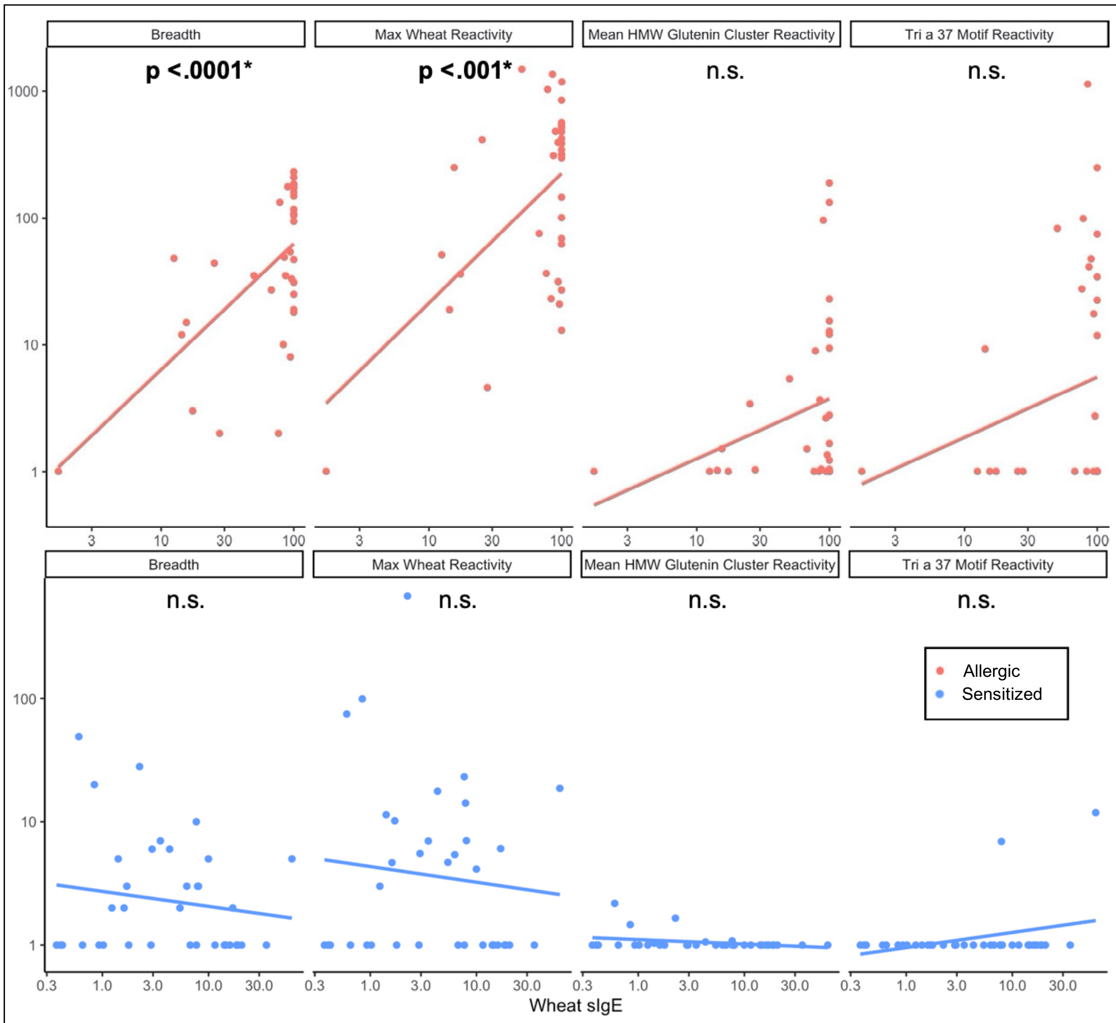

Supplemental Figure 2. Impact of Wheat Specific IgE Titer on AllerScan Results. Scatterplots comparing wheat specific IgE against overall IgE wheat breadth, maximum IgE wheat reactivity, mean IgE HMW glutenin reactivity from the cluster in Fig 4D, and IgE reactivity to the Tri a 37 epitope identified in Figure 3. Comparisons were made against all allergic (top) and sensitized (bottom) individuals. Linear regressions are shown for each plot and significance was determined by F - test.

Patients	Age at Time of Sample	Gender	Race	Ethnicity	Wheat Allergy Status	Peanut Allergy Status	Wheat IgE	Peanut IgE	Total IgE	Other food allergies	Initial WIT Treatment
Pt1	7	Female	Asian	Not Hispanic or Latino	A	A	>=100	>=100	>6000.0	Milk, Egg, Sesame, Peanut, Tree nuts, Shellfish, Soy, Wheat, lentils, sunflower seeds, garlic, banana	NA
Pt2	11	Female	White	Not Hispanic or Latino	A	A	27.6	1.5	275	Peanut, Tree nuts, Wheat, cashew, pistachio, rye, barley	NA
Pt3	12	Female	White	Not Hispanic or Latino	A	A	1.68	5.49	242	Egg, Peanut, Tree nuts, Fish, Shellfish, Wheat, pineapple	NA
Pt4	7	Male	Black/African American	Not Hispanic or Latino	A	A	96.4	60.7	23723	Milk, Egg, Peanut, Tree nuts, Fish, Shellfish, Soy, Wheat	NA
Pt5	5	Male	White	Not Hispanic or Latino	A	A	>=100	6.25	536	Concentrated Milk, Peanut, Tree nuts, Wheat, barley, rye	NA
Pt6	10	Male	Asian	Not Hispanic or Latino	S	A	61	45	8708	Milk, Egg, Sesame, Peanut, Tree nuts, Soy, Wheat, Garlic, ginger, legumes, all grains except rice	NA
Pt7	9	Male	Multiracial	Hispanic or Latino	A	A	17.3	>=100	1351	Milk, Egg, Sesame, Peanut, Tree nuts, Wheat, peas, lentils, chickpea	NA
Pt8	14	Female	White	Not Hispanic or Latino	A	A	12.5	6.81	1416	Milk, Frank Egg, Peanut, Tree nuts, Fish, Shellfish, Wheat	NA
Pt9	9	Male	White	Not Hispanic or Latino	A	S	>=100	0.75	827	Egg, Peanut, Tree nuts, Fish, Shellfish, Wheat, rye, barley	NA
Pt10	2	Male	White	Not Hispanic or Latino	A	S	>=100	0.61	661	Concentrated Egg, Concentrated Sesame, Tree nuts, Fish, Shellfish, Wheat	NA
Pt11	16	Male	Asian	Not Hispanic or Latino	A	S	83.4	2.26	1968	Milk, Egg, Tree nuts, Shellfish, Wheat, oat	NA
Pt12	12	Female	White	Hispanic or Latino	S	A	1.77	25.2	147	Egg, Peanut, Tree nuts, Shellfish	NA
Pt13	12	Female	Multiracial	Not Hispanic or Latino	N	A	<0.35	>=100	542	Milk, Egg, Sesame, Peanut, Tree nuts, Soy	NA
Pt14	7	Female	Multiracial	Hispanic or Latino	N	A	NA	2.51		Peanut	NA
Pt15	5	Male	White	Not Hispanic or Latino	N	A	0.35	10.5	396	Peanut, Tree nuts	NA
Pt16	9	Female	White	Not Hispanic or Latino	S	A	7.69	>100	>6000	Milk, Egg, Peanut, Tree nuts	NA
Pt17	11	Female	Asian	Not Hispanic or Latino	N	A	<0.35	22.4	306	Peanut, Tree nuts	NA
Pt18	10	Female	White	Not Hispanic or Latino	N	A	NA	33.2	4255	Peanut, Tree nuts, peaches	NA
Pt19	11	Female	White	Not Hispanic or Latino	N	A	<0.35	>=100	431	Peanut, Tree nuts, concentrated Soy, lentils	NA
Pt20	14	Male	White	Not Hispanic or Latino	N	A	<0.35	15.4	456	Milk, Egg, Sesame, Peanut, Tree nuts, Shellfish, Soy, red/blue dye	NA
Pt21	39	Female	White	Not Hispanic or Latino	N	N	<0.35	<0.35	57		NA
Pt22	8	Male	Black/African American	Not Hispanic or Latino	N	N	<0.35	<0.35	12.9		NA
Pt23	10	Male	Black/African American	Not Hispanic or Latino	N	N	<0.35	<0.35	3.7		NA
Pt24	11	Male	White	Hispanic or Latino	N	N	<0.35	<0.35	127		NA
Pt25	9	Male	White	Hispanic or Latino	N	N	<0.35	<0.35	15.8		NA
Pt26	14	Male	Asian	Not Hispanic or Latino	N	N	<0.35	<0.35	184		NA
Pt27	19	Female	Asian	Not Hispanic or Latino	N	N	<0.35	<0.35	1.2		NA
Pt28	47	Female	White	Hispanic or Latino	N	N	<0.35	<0.35	23.2		NA
Pt29	14	Male	White	Hispanic or Latino	N	N	<0.35	<0.35	290		NA
Pt30	22	Female	White	Not Hispanic or Latino	N	N	<0.35	<0.35	79.2		NA
Pt31	58	Female	Black/African American	Not Hispanic or Latino	N	N	<0.35	<0.35	7.8		NA
Pt32	6	Female	White	Not Hispanic or Latino	N	N	<0.35	<0.35	1.5		NA
Pt33	6	Female	White	Not Hispanic or Latino	N	N	<0.35	<0.35	1.5		NA
Pt34	15	Male	White	Not Hispanic or Latino	S	S	14.6	21.1	>6000	Tree nuts, Shellfish, Banana	NA
Pt35	55	Female	White	Not Hispanic or Latino	N	N	<0.35	<0.35	64.4		NA
Pt36	24	Female	White	Not Hispanic or Latino	N	N	<0.35	<0.35	7.4		NA
Pt37	12	Female	White	Not Hispanic or Latino	N	N	<0.35	<0.35	1.6		NA
Pt38	16	Female	White	Not Hispanic or Latino	N	N	<0.35	<0.35	32.1		NA
Pt39	6	Female	White	Not Hispanic or Latino	N	N	<0.35	<0.35	22		NA
Pt40	12	Male	White	Not Hispanic or Latino	N	N	<0.35	<0.35	7.6		NA
Pt41	15	Male	White	Not Hispanic or Latino	N	N	<0.35	<0.35	5.7		NA
Pt42	15	Male	Black/African American	Not Hispanic or Latino	N	N	<0.35	<0.35	73.3		NA
Pt43	37	Female	White	Hispanic or Latino	N	N	NA	NA	58.9		NA
Pt44	14	Male	White	Not Hispanic or Latino	N	N	<0.35	<0.35	151		NA
Pt45	47	Female	White	Not Hispanic or Latino	N	N	<0.35	<0.35	14.4		NA
Pt46	2	Male	White	Not Hispanic or Latino	N	S	<0.35	0.39	201	Egg	NA
Pt47	30	Female	White	Not Hispanic or Latino	N	N	<0.35	<0.35	7.7		NA
Pt48	24	Female	White	Not Hispanic or Latino	N	N	<0.35	<0.35	3.9		NA
Pt49	51	Female	White	Not Hispanic or Latino	N	N	<0.35	<0.35	25.2	Cashew, Brazil Nut	NA
Pt50	12	Female	Black/African American	Not Hispanic or Latino	N	N	<0.35	<0.35	9.4		NA
Pt51	12	Female	Black/African American	Not Hispanic or Latino	N	N	<0.35	<0.35	9.4		NA
Pt52	5	Female	White	Not Hispanic or Latino	N	S	<0.35	1.48	207	cashew, pistachio and mango	NA
Pt53	7	Female	White	Not Hispanic or Latino	N	S	<0.35	0.41	55.7		NA
Pt54	12	Male	White	Not Hispanic or Latino	S	S	7.89	12.4	4022	Milk, Egg, Sesame, Tree nuts, Fish, Shellfish, Chicken	NA
Pt55	11	Male	White	Not Hispanic or Latino	S	S	6.72	79.3	>6000	Egg, Peanut, Tree nuts, Soy, pork, peaches	NA
Pt56	6	Male	Black/African American	Not Hispanic or Latino	S	A	1.02	>=100	510	Milk, Egg, Peanut, Tree nuts, Fish	NA
Pt57	10	Male	White	Not Hispanic or Latino	S	A	4.3	>=100	8238	Sesame, Peanut, Tree nuts	NA
Pt58	17	Male	White	Not Hispanic or Latino	S	A	2.87	31.2	15,795	Sesame, Peanut, Tree nuts, Shellfish	NA
Pt59	12	Female	White	Not Hispanic or Latino	S	A	18.4	24.7	>6000	Milk, Egg, Peanut, Tree nuts, Fish, Shellfish	NA
Pt60	17	Male	White	Not Hispanic or Latino	S	A	0.42	>=100	451	Peanut, Tree nuts	NA
Pt61	6	Male	Black/African American	Not Hispanic or Latino	S	A	15.9	81.2	5214	Milk, Egg, Peanut, Tree nuts, Fish, Shellfish, concentrated Soy	NA
Pt62	10	Male	Multiracial	Not Hispanic or Latino	S	A	9.98	41.8	1754	Straight Milk, Sesame, Peanut, Tree nuts, Fish, Shellfish	NA
Pt63	9	Female	Asian	Not Hispanic or Latino	S	A	8.04	>=100	1854	Peanut, Tree nuts	NA
Pt64	8	Male	Asian	Not Hispanic or Latino	S	A	11.5	>100	7374	Egg, Sesame, Peanut, Tree nuts, Fish, Soy, cococnut, legumes except for green beans	NA
Pt65	10	Female	White	Not Hispanic or Latino	S	A	0.65	5.82	166	Peanut, Tree nuts, Shellfish	NA
Pt66	9	Male	White	Not Hispanic or Latino	S	A	35.1	>100	5593	Sesame, Peanut, Tree nuts, Fish, Shellfish, Wheat, Oat, Kiwi	NA
Pt67	4	Male	White	Not Hispanic or Latino	S	A	1.41	>100	730	Peanut, Tree nuts, Shellfish, Chia, flaxseed	NA
Pt68	22	Male	Multiracial	Not Hispanic or Latino	S	A	1.6	10.4	243	Peanut, Tree nuts	NA
Pt69	14	Female	White	Not Hispanic or Latino	S	A	3.52	>=100	2627	Milk, Egg, Peanut, Tree nuts	NA
Pt70	5	Female	White	Not Hispanic or Latino	S	A	18.9	>=100	22,672	Milk, Egg, Sesame, Peanut, Tree nuts, mustard	NA
Pt71	8	Male	Asian	Not Hispanic or Latino	S	A	0.4	>100	1148	Peanut, Tree nuts	NA
Pt72	5	Male	White	Not Hispanic or Latino	S	N	0.84	0.34	151		NA
Pt73	8	Female	White	Not Hispanic or Latino	S	S	1.23	0.89	389		NA
Pt74	15	Female	Black/African American	Not Hispanic or Latino	S	S	5.38	9.69	1222	Shellfish, shrimp	NA
Pt75	19	Female	Asian	Not Hispanic or Latino	S	S	1.7	1.93	621		NA
Pt76	2	Male	Multiracial	Not Hispanic or Latino	S	S	7.67	36.2	1103	Sesame, Peanut, kiwi	NA
Pt77	7	Male	White	Not Hispanic or Latino	S	S	0.6	1.18	218	Peanut, Tree nuts, Fish	NA
Pt78	13	Female	Multiracial	Not Hispanic or Latino	S	S	6.24	2.42	1652	Tree nuts, Shellfish	NA
Pt79	11	Female	Black/African American	Not Hispanic or Latino	S	S	16.9	27.5	2956	Peanut, Tree nuts, Fish, Shellfish	NA
Pt80	38	Female	Multiracial	Not Hispanic or Latino	S	S	0.93	2.98	623		NA
Pt81	11	Male	Multiracial	Hispanic or Latino	S	S	2.94	8.57	1882	Shellfish	NA
Pt82	15	Male	White	Not Hispanic or Latino	S	S	2.24	2.73	464	Shellfish	NA
Pt83	5	Male	Asian	Not Hispanic or Latino	S	S	14.1	36.1	4992	Straight Egg, Peanut, Tree nuts, Fish	NA
Pt84	38	Male	White	Not Hispanic or Latino	S	S	0.37	0.58	970		NA
Pt85	15	Male	White	Not Hispanic or Latino	S	S	20.5	37	41,812	Egg, Soy	NA
FA1JH001*	10	male	white	Not Hispanic or Latino	A		90		209	PEANUT, TREENUT, BARLEY	Placebo
FA1JH002*	16	male	white	Not Hispanic or Latino	A	S	101	N/A	369	BARLEY, RYE, OAT	Wheat
FA1JH003*	8	male	white	Not Hispanic or Latino	A	N/A	50.1	N/A	336	BARLEY, RYE, TREE NUTS	Wheat
FA1JH004*	6	male	Asian	Not Hispanic or Latino	A	A	25.2	66.7	281	EGG, GARLIC,TREE NUT,PEANUT,MILK	Placebo
FA1JH005*	11	male	White	Not Hispanic or Latino	A	A	101	48.5	2244	MILK, SES, TN, PN, EGG, BARLEY	Wheat
FA1JH006*	7	male	Asian	Not Hispanic or Latino	A	N/A	101	N/A	2598	MILK, EGGS, NUTS, LENTILS,	Placebo
FA1JH007*	11	male	White	Not Hispanic or Latino	A	A	86.8	13	985	EGG, PN, TN, BARLEY	Placebo
FA1JH008*	7	female	White	Not Hispanic or Latino	A	A	94.1	94	594	MILK, WHEAT, EGG, PN, TN, SESAME, GREENBEANS	Wheat
FA1JH009*	7	female	Asian	Not Hispanic or Latino	A	A	94.3	15.6	999	MILK, EGG, SOY, PEANUT, TREENUTS, SHELLFISH, OAT, GARLIC, SEASONAL, AND MUSTARD	Placebo
FA1JH010*	11	male	White	Not Hispanic or Latino	A	A	67.8	100	754	PEANUTS, TREENUTS, SESAME, CHICKPEAS, LENTILS, BARLEY, OAT, BUCKWHEAT	Placebo
FA1JH011*	9	male	White	Not Hispanic or Latino	A	A	101	8.14	249	EGG, PEANUT, SOY, BARLEY, RYE	Wheat
FA1JH012*	17	male	Black/African American	Not Hispanic or Latino	A	A	76.7	unknown	1391	MILK, PN, TN, COCONUT, SESAME	Wheat
FA1JH013*	5	male	Asian	Not Hispanic or Latino	A	A	101	20.7	336	BARLEY, EGG, PN	Placebo
FA1JH014*	4	male	Asian	Not Hispanic or Latino	A	A	101	15.3	628	TREE NUTS, PEANUTS	Placebo
FA1JH015*	4	male	White	Not Hispanic or Latino	A	A	101	79.7	577	MILK, PEANUT, TREE NUT, EGG	Placebo
FA1MS003*	22	Male	White	Not Hispanic or Latino	A	No	84.9	unk	200	BARLEY, RYE, OAT	Placebo
FA1MS004*	7	Female	White	Not Hispanic or Latino	A	No	101	unk	1357	E, BARLEY, RYE, TN, LENTIL, F	Wheat
FA1MS005*	11	Female	White	Not Hispanic or Latino	A	no	101	unk	1322	BARLEY, RYE, OAT	Placebo
FA1MS006*	10	Female	White	Not Hispanic or Latino	A	Yes	14.3	unk	706	SOY, MILK, E, PN, TN, SF	Wheat
FA1MS008*	6	Female	White	Not Hispanic or Latino	A	Yes	101	unk	1669	SOY, TN, PN, SEEDS, SESAME	Wheat
FA1MS009*	13	Male	White	Not Hispanic or Latino	A	No	78.5	unk	267	MILK, EGGS, SESAME, BARLEY, OA	Placebo
FA1MS010*	10	Male	White	Not Hispanic or Latino	A	No	101	unk	380	RYE, BARLEY, WALNUT, HAZELNUT	Wheat
FA1MS011*	4	Male	White	Not Hispanic or Latino	A	Yes	101	15.3	2482	MILK, E, PN, TN, SF, SESAME	Placebo
FA1MS012*	13	Male	White	Not Hispanic or Latino	A	Yes	15.5	<0.35	276	UNBAKED MILK, EGG, RYE, BARLEY	Wheat
FA1MS013*	17	Male	White	Not Hispanic or Latino	A	Yes	101	20.5	672	PN, T N, SESAME, BARLEY, FISH	Wheat

Supplemental Table 1 – Table of clinical metadata describing study participants. Table lists race, gender, age at time of sample, wheat and peanut allergy status, wheat, peanut and total IgE levels and known other food allergies

Group	Organism	N
Vertebrate	Domestic water buffalo	5
Vertebrate	Sheep	11
Vertebrate	Bovine	24
Vertebrate	Chicken	11
Invertebrate	Syntelopodeuma_sp.	5
Invertebrate	Scolopendra_sp.	8
Invertebrate	Sea spider	5
Invertebrate	Lepeophtheirus_salmonis	9
Invertebrate	Glossina_morsitans	7
Invertebrate	Fruit fly	11
Invertebrate	Southern house mosquito	5
Invertebrate	Yellowfever mosquito	9
Invertebrate	Asian swallowtail butterfly	5
Invertebrate	Silk moth	6
Invertebrate	Pink hibiscus mealybug	7
Invertebrate	American cockroach	5
Invertebrate	brown sailor spider	6
Invertebrate	Dermanyssus_gallinae	5
Invertebrate	Mold mite	5
Invertebrate	Mite	5
Invertebrate	Haemaphysalis_qinghaiensis	6
Invertebrate	Brown ear tick	6
Invertebrate	Atlantic horseshoe crab	6
Invertebrate	Antarctic krill	5
Invertebrate	South African spiny lobster	7
Invertebrate	Chinese spiny lobster	7
Invertebrate	Red king crab	7
Invertebrate	Razor clam	8
Grains	Tausch's goatgrass	6
Grains	Triticum_spelta	6
Grains	Wheat	638
Grains	Hordeum_vulgare	9
Grains	Oat	11
Legumes + Tree nuts	Garden pea	20
Legumes + Tree nuts	Narrow-leaved blue lupine	17
Legumes + Tree nuts	White lupine	10
Legumes + Tree nuts	Lentil	6
Legumes + Tree nuts	Soybean	64
Legumes + Tree nuts	Chickpea	5
Legumes + Tree nuts	Wild peanut	6
Legumes + Tree nuts	Peanut	75
Legumes + Tree nuts	White mustard	5
Legumes + Tree nuts	Pecan	12
Legumes + Tree nuts	English walnut	9
Legumes + Tree nuts	European hazel	8
Legumes + Tree nuts	Prunus_dulcis	7
Legumes + Tree nuts	Bacillus_clausii	5

Supplemental Table 2 – Reference table of group memberships in Fig 2. Column 1 indicates group name, column 2 indicates the organism, and column 3 indicates the number of peptides shown in Fig 2 for each organism.
