# Supplementary material for: Profiling serum antibodies with a pan allergen phage library identifies key wheat allergy epitopes
Source: Nat Commun. 2021 Jan 22;12:379. doi: 10.1038/s41467-020-20622-1 (PMC7822912; doi:10.1038/s41467-020-20622-1)
Supplement: Supplementary file 3 — Reporting Summary [file 41467_2020_20622_MOESM3_ESM.pdf]

nature research

Corresponding author(s): Harry Larman

Last updated by author(s): Nov 16, 2020

Reporting Summary

Nature Research wishes to improve the reproducibility of the work that we publish. This form provides structure for consistency and transparency in reporting. For further information on Nature Research policies, see our Editorial Policies and the Editorial Policy Checklist.

Statistics

For all statistical analyses, confirm that the following items are present in the figure legend, table legend, main text, or Methods section.

n/a	Confirmed	
☐	☒	The exact sample size (*n*) for each experimental group/condition, given as a discrete number and unit of measurement
☐	☒	A statement on whether measurements were taken from distinct samples or whether the same sample was measured repeatedly
☐	☒	The statistical test(s) used AND whether they are one- or two-sided<br>*Only common tests should be described solely by name; describe more complex techniques in the Methods section.*
☐	☒	A description of all covariates tested
☐	☒	A description of any assumptions or corrections, such as tests of normality and adjustment for multiple comparisons
☒	☐	A full description of the statistical parameters including central tendency (e.g. means) or other basic estimates (e.g. regression coefficient) AND variation (e.g. standard deviation) or associated estimates of uncertainty (e.g. confidence intervals)
☐	☒	For null hypothesis testing, the test statistic (e.g. *F*, *t*, *r*) with confidence intervals, effect sizes, degrees of freedom and *P* value noted<br>*Give P values as exact values whenever suitable.*
☒	☐	For Bayesian analysis, information on the choice of priors and Markov chain Monte Carlo settings
☒	☐	For hierarchical and complex designs, identification of the appropriate level for tests and full reporting of outcomes
☒	☐	Estimates of effect sizes (e.g. Cohen's *d*, Pearson's *r*), indicating how they were calculated

Our web collection on statistics for biologists contains articles on many of the points above.

Software and code

Policy information about availability of computer code

Data collection	All code used to generate data was implemented in R v.3.6.1. We utilized the R packages, Rsubread (v.2.4.1) and Shortread (v.1.48.0), to map sequencing reads to the Allergome library. Next, we utilized the edgeR R (v.3.32.0) software package to determine which peptide reactivities were significantly enriched.
Data analysis	All analysis was developed and implemented in R v.3.6.1. All heatmaps were constructed using the pheatmap package (v.1.0.12) and all network graph analyses utilized the igraph software package (v.1.2.4.2); all other plots were built using the tidyverse package suite (v.1.3.0). All performed statistical tests were two-sided Wilcoxon signed-rank tests unless otherwise specified. All custom code generated during this study can be accessed at https://github.com/drmonaco/AllerScan.

For manuscripts utilizing custom algorithms or software that are central to the research but not yet described in published literature, software must be made available to editors and reviewers. We strongly encourage code deposition in a community repository (e.g. GitHub). See the Nature Research guidelines for submitting code & software for further information.

Data

Policy information about availability of data

All manuscripts must include a data availability statement. This statement should provide the following information, where applicable:

- Accession codes, unique identifiers, or web links for publicly available datasets
- A list of figures that have associated raw data
- A description of any restrictions on data availability

The data that support the findings of this study are available in figshare with the identifier [https://doi.org/10.6084/m9.figshare.13315220.v1]. All source data can be generated by running analyzing the publicly hosted dataset via through the custom scripts at https://github.com/drmonaco/AllerScan. The initial Allergome database used for the construction of Allergome phage library can be found at https://www.uniprot.org/uniprot/?query=database%3A%28type%3Aallergome%

29&sort=score

Field-specific reporting

Please select the one below that is the best fit for your research. If you are not sure, read the appropriate sections before making your selection.

- [x] Life sciences
- [ ] Behavioural & social sciences
- [ ] Ecological, evolutionary & environmental sciences

For a reference copy of the document with all sections, see nature.com/documents/nr-reporting-summary-flat.pdf

Life sciences study design

All studies must disclose on these points even when the disclosure is negative.

Sample size	We did not perform sample-size calculations; instead the size of the study was largely determined by the availability of serum samples. The studies composition was designed to profile anti-allergen serum antibodies, and to determine differences in the anti-allergen antibody profiles of allergic and sensitized individuals. Nonetheless, we found significant differences between different subpopulations (and compelling overlaps within the same subpopulations) suggesting ample sampling occurred.
Data exclusions	Seven samples were excluded from further analysis for failing quality control; each showing artifactual reactivities to a large fraction of the library. These samples were excluded prior to the unblinding of the data and failed our internal qc standards that were not specific to this study.
Replication	For each plate at minimum 3 samples were randomly selected to be screened in replicate. Prior to unblinding, all replica pairs were compared and we found high concordance between pairs (R2 > 0.9). One example of a replica pair is shown Fig 1e, other pairs can be shown upon request.
Randomization	Samples were added randomly to two 96 well plates prior to data collection for IgE and IgG testing respectively. Each sample was given a random well and plate position. Sample placement was randomized for IgG testing and was re-randomized for IgE testing.
Blinding	Investigators who performed the AllerScan assay were blinded to the wheat and peanut allergy status (and all other patient data) of samples until data collection was complete. Investigators were blinded to outcomes and placebo/treatment status of wheat oral immunotherapy trial samples during data analysis.

Reporting for specific materials, systems and methods

We require information from authors about some types of materials, experimental systems and methods used in many studies. Here, indicate whether each material, system or method listed is relevant to your study. If you are not sure if a list item applies to your research, read the appropriate section before selecting a response.

Materials & experimental systems

n/a	Involved in the study	
[ ]	[x]	Antibodies
[x]	[ ]	Eukaryotic cell lines
[x]	[ ]	Palaeontology and archaeology
[x]	[ ]	Animals and other organisms
[ ]	[x]	Human research participants
[ ]	[x]	Clinical data
[x]	[ ]	Dual use research of concern

Methods

n/a	Involved in the study	
[x]	[ ]	ChIP-seq
[x]	[ ]	Flow cytometry
[x]	[ ]	MRI-based neuroimaging

Antibodies

Antibodies used	To quanitfy patient IgG serum concentrations we used a goat anti-human IgG-HRP antibody (Southern Biotech, cat - 2040-05)<br>To immunocapture patient IgE antibodies we used biotinylated omalizumab (Labome - KBI1021)
Validation	Antibody 2040-05 was validated by the manufacturer for specific binding with the heavy chain of human IgG for use in ELISAs. Omalizumab (is a therapeutic monoclonal anti-IgE antibody that has a well-characterized efficacy and safety profile in patients with asthma. Further information can be found at https://www.accessdata.fda.gov/drugsatfda_docs/label/2003/omalgen062003LB.pdf

Human research participants

Policy information about studies involving human research participants

Population characteristics	Sera screened for this study came from two sources. The first set of sera were obtained from 58 patients with IgE-mediated food allergy along with 25 age-matched healthy controls who were enrolled on a Natural History of Food Allergy protocol at the National Institutes of Health. Patients were characterized as wheat and/or peanut allergic/sensitized/non-allergic. Allergy status was determined by a combination of convincing patient history and ImmunoCAP IgE testing. The second set of sera came from patients enrolled in a randomized, double-blind, placebo-controlled wheat oral immunotherapy trial. These patients had confirmed wheat allergy by a initial positive double-blind, placebo-controlled wheat oral food challenge. Samples were age and gender matched in this study.
Recruitment	Patients screened in this study were either enrolled on a Natural History of Food Allergy protocol at the National Institutes of Health or were enrolled in a randomized, double-blind, placebo-controlled wheat oral immunotherapy trial at Johns Hopkins University and the Icahn School of Medicine at Mount Sinai in New York. All patient's enrolled in this trial had moderate/ severe wheat allergy that may be more severe than wheat allergy at large in the US.
Ethics oversight	Samples came from the National Institutes of Health, Johns Hopkins University and the Icahn School of Medicine at Mount Sinai in New York. All three institutional review boards approved their respective study protocols.

Note that full information on the approval of the study protocol must also be provided in the manuscript.

Clinical data

Policy information about clinical studies

All manuscripts should comply with the ICMJEguidelines for publication of clinical research and a completedCONSORT checklist must be included with all submissions.

Clinical trial registration	NCT01980992
Study protocol	Full protocol can be found at https://www.jacionline.org/action/showPdf?pii=S0091-6749%2818%2931294-6
Data collection	All data collection information can be found at https://clinicaltrials.gov/ct2/show/study/NCT01980992 and https://www.jacionline.org/action/showPdf?pii=S0091-6749%2818%2931294-6
Outcomes	Trial outcomes are defined at https://clinicaltrials.gov/ct2/show/study/NCT01980992
